# Temporal stability of chimpanzee social culture

**DOI:** 10.1098/rsbl.2021.0031

**Published:** 2021-05-26

**Authors:** Edwin J. C. van Leeuwen

**Affiliations:** ^1^ Department for Comparative Cultural Psychology, Max Planck Institute for Evolutionary Anthropology, Deutscher Platz 6, 04103, Leipzig, Germany; ^2^ Behavioral Ecology and Ecophysiology Group, Department of Biology, University of Antwerp, Universiteitsplein 1, 2610 Wilrijk, Belgium; ^3^ Centre for Research and Conservation, Royal Zoological Society of Antwerp, Koningin Astridplein 26, 2018, Antwerp, Belgium

**Keywords:** chimpanzees, social culture, temporal stability, grooming handclasp

## Abstract

Culture is a hallmark of the human species, both in terms of the transmission of material inventions (e.g. tool manufacturing) and the adherence to social conventions (e.g. greeting mannerisms). While material culture has been reported across the animal kingdom, indications of social culture in animals are limited. Moreover, there is a paucity of evidencing cultural stability in animals. Here, based on a large dataset spanning 12 years, I show that chimpanzees adhere to arbitrary group-specific handclasp preferences that cannot be explained by genetics or the ecological environment. Despite substantial changes in group compositions across the study period, and all chimpanzees having several behavioural variants in their repertoires, chimpanzees showed and maintained the within-group homogeneity and between-group heterogeneity that are so characteristic of the cultural phenomenon in the human species. These findings indicate that human culture, including its arbitrary social conventions and long-term stability, is rooted in our evolutionary history.

## 1. Introduction

Humans’ aptitude to acquire novel solutions to pervasive challenges derives not only from an astute innovative capability, but also from high-fidelity social learning [1]—a dual engine radiating in a plethora of cultural phenotypes, arguably unparalleled in the animal kingdom [2–4]. Culture—defined as group-specific behavioural patterns that have come about by means of social learning [5]—represents material advantages in the form of short-cut access to innovations, like the manufacturing of blade tools made from bone [6], but also social conventions that are inherently shared, like wearing pendants made from teeth [6], which might spur social bonding or symbolize group-identity [2]. Rudimentary forms of material culture have been thoroughly evidenced in several non-human animal (henceforth ‘animal’) species (e.g. [7,8]), but the identification of animal *social* culture remains limited to short cross-sectional reports of group differences in social behaviour [9–11]. Moreover, while typical for human culture and relevant to the study of cultural evolution [12,13], there is a paucity with respect to reporting temporal stability of cultural traditions in animals. Social games in capuchin monkeys (*Cebus capucinus* [14]) and stone-handling behaviours in Japanese macaques (*Macaca fuscata* [15]) have been found to exhibit stability, albeit with irregular infusions of new variations (also see [16]). In the domain of functional communication, evidence for both rapid song revolution (humpback whales (*Megaptera novaeangliae*) [17]; white-crowned sparrows (*Zonotrichia leucophrys pugetensis*) [18]) and temporal stability of the acoustic structure (humpback whales [19]; sperm whales (*Physeter microcephalus*) [20]; swamp sparrows (*Melospiza georgiana*) [21]) has been documented. Such findings provide impetus to take seriously the workings of cultural evolution in animal species [12,13,22].

Here, I provide evidence for the temporal stability of the first arbitrary social custom ever described in chimpanzees (*Pan troglodytes*): the grooming handclasp (GHC) [11; Video 1]. Building on the original [11] and subsequent reports establishing the cultural nature of the handclasp [23–25], I studied group differences in style variation longitudinally and found that despite changing group compositions due to births and deaths, the specific pattern of the handclasp custom in chimpanzees remains highly group-specific. Cultural novices (mostly juveniles maturing into the more serious social dynamics of adult chimpanzees, including grooming and coalition formation [26,27]) predominantly adopted their group-specific handclasp variant. These findings presume the workings of behavioural mechanisms that maintain group-level signatures across time in chimpanzees [28] and identify the aptitude to persist in arbitrary socio-cultural conventions as an evolutionary foundation of human nature.

## 2. Material and methods

### (a) Subjects and study site

Subjects were 71 chimpanzees (*Pan troglodytes schweinfurthii*)^1^ from Group 1 (13 females/10 males) and Group 2 (32 females/16 males) at the Chimfunshi Wildlife Orphanage Trust, Zambia (see electronic supplementary material, table S1 for demographic details). These chimpanzees live in large (160–190 acres) forested enclosures consisting of miombo vegetation closely resembling chimpanzees' natural habitat [29]. Chimpanzees at Chimfunshi engage in fission–fusion dynamics, encounter large (bush buck) and dangerous (poisonous snakes) animals in their enclosure, and sleep outside. The chimpanzees are supplementary fed twice a day, during which most of them are visible for observers. Outside the feeding windows, the majority of chimpanzees are also regularly visible as seen from elevated surfaces and the fence line.

### (b) Data collection and coding

Data were collected by means of all-occurrence sampling in 2007, 2010, 2011, 2017 and 2019. The GHC was operationalized as a symmetrical postural configuration in which ‘each of the participants simultaneously extends an arm overhead and then either one clasps the other's wrist or hand, or both clasp each other's hand’ [11, p. 238]. To scrutinize behavioural variants, two more clasping styles were operationalized, making for four variations: palm, wrist, forearm or other [24]. All combinations of styles were observed at least once across groups. The category ‘other’ included styles that could not be reliably classified as either palm, wrist or forearm, but were too diffuse to form one distinct category (e.g. elbow, upper arm). Grooming bouts could contain more than one GHC (*n* = 205), where a grooming bout was defined as two individuals being in close contact of whom at least one engaged in social grooming; the start was defined by the first grooming action, the end by none of the partners grooming for at least 30 s. Due to a short sampling window in 2017 resulting in few observed GHC bouts, data from 2017 were collated with 2019.

### (c) Analysis

**Figure 1.**
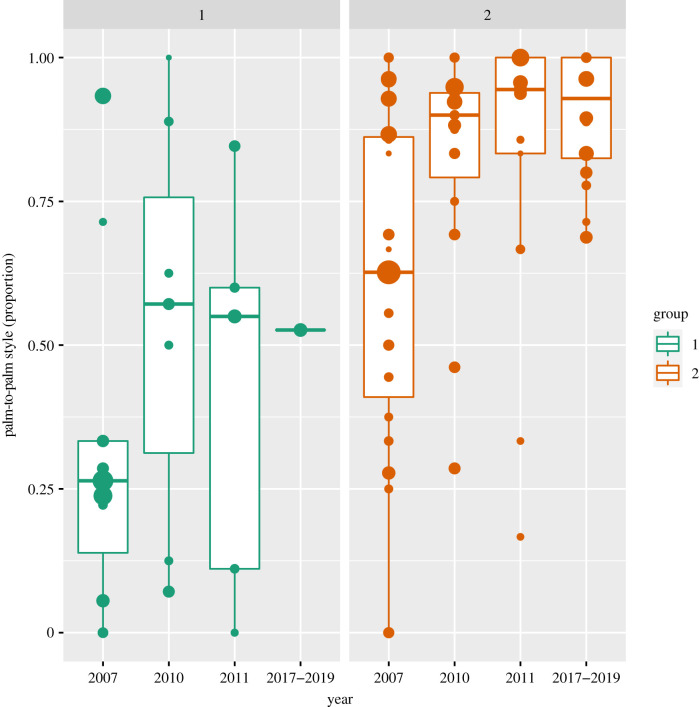
GHC style preferences (palm-to-palm, *y*-axis) show long-term stability (time in years, *x*-axis) of group differences (left panel: Group 1, right panel: Group 2) in semi-wild chimpanzees. Dots represent mean dyadic preferences scaled to frequency of interaction (range 5–75), medians are represented by the bold, horizontal lines within the boxes. The boxes represent the interquartile range (IQR), the vertical lines attached to the boxes represent Q1–1.5 IQR (lower) and Q3 + 1.5 IQR (upper).

I analysed the stability of group differences in GHC style by applying generalized linear mixed models in the R statistical environment (v. 4.0.2) using the ‘lme4’ package [30]. Palm-to-palm style (yes/no) was modelled as a function of group, year (including their interaction), dyad-sex combination (MM–FM–FF) and the absolute age difference between both partners (*z*-transformed) as fixed effects, and both partners' identity, dyad, date and grooming bout as random effects with a binomial model with logit link function [31]. Years of data collection (dummy coded) were entered as random slope components in both partners’ identity and dyad [32]. Temporal stability and group differences in handclasp style were assessed with model comparisons [31,33] by testing the group–year interaction and the main effect of group, respectively. Confidence intervals for the group parameter were calculated with the profile likelihood method from the ‘lme4’ package ([30]; function ‘confint.merMod’) and with the ‘emmeans' package [34] for assessing post hoc contrasts within the levels of the dyad-sex parameter. Following precedents in the handclasp literature [25,35,36] and because it was the most frequently observed handclasp style, I focused on *palm* clasping (for corroborating analyses on *wrist* clasping, see electronic supplementary material). Given the interactive nature of GHC and in line with previous GHC style assessments [24,25], I modelled dyadic instead of individual style preferences. The full model provided a better fit to the data than an intercept only model (likelihood ratio test [33]: *χ*^2^ = 74.52, d.f. = 12, *p* < 0.0001). Parameter metrics were obtained with the ‘drop1’ (for *p*-values) and ‘summary’ functions (for estimates). I analysed all data (Group 1: *N*_bouts_ = 560, *N*_dyads_ = 26; Group 2: *N*_bouts_ = 1489, *N*_dyads_ = 229; [37]), and a subset of the data with only those dyads included that engaged in GHC at least five times (to optimize tapping into *preferences*), yielding qualitatively similar results (Group 1: *N*_bouts_ = 455, *N*_dyads_ = 26; Group 2: *N*_bouts_ = 1182, *N*_dyads_ = 73). To present preferences rather than chance observations and to remain consistent with previous literature [24,38], I plotted the data including dyads that engaged in GHC at least five times (figure 1).

## 3. Results

Palm-to-palm clasping was substantially more pronounced in Group 2 compared to Group 1 across all years (likelihood ratio test (LRT) main effect group: *χ*^2^ = 9.20, d.f. = 1, *p* = 0.0031; estimate ± s.e. = 1.08 ± 0.36, 95% CI: 0.31–1.81). The odds for chimpanzees in Group 2 to engage in palm-to-palm clasping were 2.93× larger than for the chimpanzees in Group 1 (mean ±s.d. Group 1: 44.64 ± 49.75%; Group 2: 77.57 ±41.72%). Individual-level palm preferences were consistent with the group differences in dyadic preferences (mean ± s.d. Group 1: 53.4 ± 30.4%; Group 2: 78.74 ± 20.93%). The direction and magnitude of this group-level variation did not obviously change over a 12-year time period (LRT interaction group x year: *χ*^2^ = 4.27, d.f. = 4, *p* = 0.37). For similar findings on *wrist* usage during handclasping, see electronic supplementary material, figure S1. Jackknife resampling of the GLMMs (dyad omissions with replacement) revealed that the groups reliably (estimate range of the main effect of group: 1.00–1.41) and stably (mean ± s.d. *p*-value interaction parameter group x year: 0.367 ± 0.028) differed in their palm-to-palm preferences (figure 1).

Female–female dyads were more likely to engage in palm-to-palm clasping (back-transformed probability = 0.74, 95% CI: 0.59–0.84) than other dyad-sex combinations (LRT: *χ*^2^ = 6.96, d.f. = 2, *p* = 0.031; FM dyads = 0.58, 95% CI: 0.43–0.71; MM dyads = 0.58, 95% CI: 0.35–0.79; electronic supplementary material, table S2). Yet, the identified group differences in palm-to-palm clasping were not obviously different for the dyad-sex combinations (post hoc LRT interaction group × dyad-sex combination: *χ*^2^ = 1.82, d.f. = 2, *p* = 0.403) and were observed for each of the dyad-sex combinations (electronic supplementary material, figure S2 and table S2), also for *wrist* clasping (electronic supplementary material, figure S3 and table S3). Absolute age difference between the GHC partners did not affect the probability to engage in palm-to-palm clasping (*χ*^2^ = 0.53, d.f. = 1, *p* = 0.535).^2^

**Figure 2.**
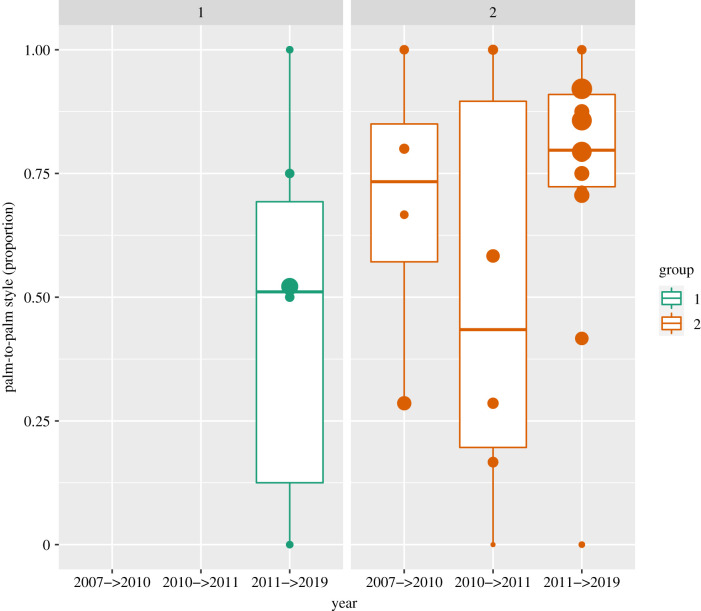
GHC style preferences (palm-to-palm, *y*-axis) of new adopters across the study period (time in years T_from_ —> T_to_, *x*-axis). Dots represent mean dyadic preferences scaled to frequency of interaction (range 1–38), medians are represented by the bold, horizontal lines within the boxes. The boxes represent the IQR, the vertical lines attached to the boxes represent Q1–1.5 IQR (lower) and Q3 + 1.5 IQR (upper).

Across the sampling periods, 20 chimpanzees were ‘replaced’ by 23 new GHC adopters (electronic supplementary material, table S4). Despite these substantial changes in group composition, the group differences in style preferences remained (figure 1). In more detail, the new adopters (mostly juveniles: mean age ± s.d. (in years, months) = 9.4 ± 2.1; range = 5–16 years) roughly matched their group-specific palm-to-palm preference on their first sampling year (mean ± s.d. Group 1: 46.20 ± 40.11%, Group 2: 68.38 ± 32.17%; figure 2).

## 4. Discussion

For a behaviour to be labelled ‘cultural’, scholars typically evaluate the behaviour for its (i) emergence through social learning, (ii) sharedness among group members (and absence or difference for members of other groups) and (iii) longevity [14,39,40]. While a plethora of studies have documented socially learned behavioural traditions in animals [41,42], to my knowledge, there is a paucity with respect to evidencing the stability of traditions.

One established example of cultural persistence in animals concerns tool use in wild chimpanzees: over a period of 25 years, group differences with respect to tool-material selection for nut cracking remained highly similar, despite a large number of (female) migrations [43,44]. Material culture has plausibly been part of chimpanzees' repertoire for thousands of years [45], yet social culture remains to be systematically documented in animals. Recently, cultural transmission of social customs has been suggested for traditions in great apes ([9,10,46]; also see [14,47]). Moreover, social culture in terms of socially learned patterns of association and interaction that result in group-specific sociality has been implied as an explanatory mechanism for non-random social dynamics in animals (e.g. [48–54]). In these studies, some forms of stability were documented—for instance, neighbouring groups of meerkats (*Suricata suricatta*) differed consistently in the time of day on which they emerged from their burrows across a decade [54], and reef sharks (*Carcharhinus amblyrhynchos*) were found to gather in the same close association cliques annually across four consecutive years, presumably to benefit from public information regarding food patches [52]. Arguably the best-documented case of social culture in animals comes from observations on troops of wild olive baboons (*Papio anubis*) living in the Masai Mara Reserve of Kenya. Owing to the simultaneous deaths of a substantial number of aggressive adult males (caused by a selective outbreak of tuberculosis), the respective ‘Forest Troop’ became a group of relatively socially tolerant and non-aggressive members in comparison to another group living in the same reserve (Talek troop) and the Forest Troop pre-dating the deaths [48,53]. Despite a substantial influx of new group members, across a period of at least 10 years, the Forest Troop remained markedly characterized by what the authors called a culture of ‘pacifism’—relatively high rates of grooming and affiliation, relaxed dominance hierarchies and an overall tendency of non-aggressive interactions, even between resident females and newly immigrated males [48].

Here, I report the multiyear (12 years) stability of a *variational* cultural practice [24], which is plausibly devoid of any ecological relevance. Whereas material cultures [7,55], but also culturally induced social foraging [52] and dyadic interactions [48,53] are behaviours with clear adaptive value [56], the precise variant by which chimpanzees engage in the handclasp grooming does not bear any relevance to survival or fitness. As such, stability in variant preference might be even less expected given the lack of eliciting affordances in the environment (e.g. the presence of termites might (re-)trigger termite fishing). In analogy to human culture: whereas the motivation for bridging social distances gives rise to greeting behaviours universally, the exact manner in which the greeting gesture emerges (and perpetuates) is highly culture specific [57,58]. The here reported group differences are also difficult to explain based on genetics: the study groups do not systematically differ in their subspecies composition, and whereas the handclasp behaviour *an sich* could be hypothesized to be under positive genetic selection [59] (i.e. its function is still largely unknown), the relative style preferences by which the groups choose to handclasp seem harder to place in such a selectionist framework (also see [11,24]). The finding that female dyads engaged more in palm clasping while male dyads engaged more in wrist clasping could be due to the fact that chimpanzee males may use the handclasp as a means to confirm or assert dominance over the partner. The subject's wrist position allows the partner to support the weight of the subject's clasping arm, which can be viewed as a prosocial act by the partner [11]. Albeit plausible, more research is needed to investigate this conjecture, including how such configurations are initiated.

Between-group heterogeneity is expected to gradually transition toward homogeneity owing to factors like drift, the natural cycle of births and deaths, and migrations, unless there are mechanisms in place to prevent this, like in humans [2]. Despite such changes of group composition in the studied chimpanzees, the group-specific variant preferences remained, suggesting the workings of stability-fostering mechanisms (also see [43,60]). One potent mechanism promoting group-level cultural stability is conformity—the tendency to copy the behaviour of the majority [2,28]. Whether chimpanzees are conformists remains disputed [43,61–64]—yet, the findings of this study warrant scrutiny of any chimpanzee behaviour that could bolster within-group cultural homogeneity across extended periods of time. In any case, where the minimal genetic and environmental variation across groups allows for inferring the cultural nature of the handclasp styles by means of the method of exclusion (also see [24,25]), the observed temporal stability of group-specific style preferences despite substantial population turnover provides a positive indication of the cultural hypothesis.

Recapitulating, chimpanzees retained group-specific grooming style preferences across a 12-year study period in which a substantial number of individuals replaced original group members owing to births, deaths and translocations. This stability of cultural variants indicates that (i) preliminary findings on social culture in chimpanzees are robust, (ii) animals can develop and maintain cultural preferences in the domain of arbitrary, non-fitness-related phenomena, much like the human species and (iii) animal cultures can possess the necessary ingredients in terms of variant adherence and longevity to be a potent force in gene–culture coevolutionary dynamics, thus shaping both phenotypes and genotypes in animals [12,13].

## Endnotes

1 Except for one male in Group 1 (*Pan troglodytes verus*) and one female in Group 2 (*Pan troglodytes troglodytes*). The male engaged predominantly in palm-to-palm GHC (75%; *n* = 64), the female was only observed to engage in GHC twice (50% palm-to-palm). In conjunction, these observations do not make a plausible case for GHC style preference in chimpanzees being under genetic (at the subspecies level) control.

2 Where one might expect the likelihood of palm-to-palm clasping to be higher in chimpanzees of similar ages because of their similar body sizes, we note that assessments of the effect of arm-length differentials between the clasping partners on the symmetry of handclasp style did not find evidence for this relationship either [24].

## Data accessibility

van Leeuwen, Edwin J. C. (2021), Data from: Temporal stability of chimpanzee social culture, Dryad, Dataset, https://doi.org/10.5061/dryad.6wwpzgmxh [37].

## References

[1] Heyes CM. 1994 Social-learning in animals—categories and mechanisms. Biol. Rev. Camb. Phil. Soc. **69**, 207-231. (10.1111/J.1469-185x.1994.Tb01506.X) 8054445

[2] Richerson PJ, Boyd R. 2005 Not by genes alone: how culture transformed human evolution. Chicago, IL: The University of Chicago Press.

[3] Boyd R, Richerson PJ, Henrich J. 2011 The cultural niche: why social learning is essential for human adaptation. Proc. Natl Acad. Sci. USA **108**, 10 918-10 925. (10.1073/Pnas.1100290108) PMC3131818 21690340

[4] Tennie C, Call J, Tomasello M. 2009 Ratcheting up the ratchet: on the evolution of cumulative culture. Phil. Trans. R. Soc. B **364**, 2405-2415. (10.1098/rstb.2009.0052) 19620111 PMC2865079

[5] McGrew WC. 2004 The cultured chimpanzee: reflections on cultural primatology. Cambridge, UK: Cambridge University Press.

[6] Hublin J-J et al. 2020 Initial Upper Palaeolithic *Homo sapiens* from Bacho Kiro Cave, Bulgaria. Nature **581**, 299-302. (10.1038/s41586-020-2259-z) 32433609

[7] McGrew WC. 1992 Chimpanzee material culture: implications for human evolution. Cambridge, UK: Cambridge University Press.

[8] Hunt GR. 1996 Manufacture and use of hook-tools by New Caledonian crows. Nature **379**, 249-251. (10.1038/379249a0)

[9] van Leeuwen EJC, Staes N, Verspeek J, Hoppitt WJE, Stevens JMG. 2020 Social culture in bonobos. Curr. Biol. **30**, R261-R262. (10.1016/j.cub.2020.02.038) 32208147

[10] Nakamura M, McGrew WC, Marchant LF, Nishida T. 2000 Social scratch: another custom in wild chimpanzees? Primates **41**, 237-248. (10.1007/BF02557594) 30545176

[11] McGrew WC, Tutin CEG. 1978 Evidence for a social custom in wild chimpanzees? Man **13**, 234-251. (10.2307/2800247)

[12] Whitehead H, Laland KN, Rendell L, Thorogood R, Whiten A. 2019 The reach of gene–culture coevolution in animals. Nat. Commun. **10**, 2405. (10.1038/s41467-019-10293-y) 31160560 PMC6546714

[13] Whiten A. 2019 Cultural evolution in animals. Annu. Rev. Ecol. Evol. Syst. **50**, 27-48. (10.1146/annurev-ecolsys-110218-025040)

[14] Perry S et al. 2003 Social conventions in wild white-faced capuchin monkeys—Evidence for traditions in a neotropical primate. Curr. Anthropol. **44**, 241-268. (10.1086/345825)

[15] Huffman MA, Nahallage CAD, Leca J-B. 2008 Cultured monkeys: social learning cast in stones. Curr. Dir. Psychol. Sci. **17**, 410-414. (10.1111/j.1467-8721.2008.00616.x)

[16] Schofield DP, McGrew WC, Takahashi A, Hirata S. 2018 Cumulative culture in nonhumans: overlooked findings from Japanese monkeys? Primates **59**, 113-122. (10.1007/s10329-017-0642-7) 29282581 PMC5843669

[17] Noad MJ, Cato DH, Bryden MM, Jenner M-N, Jenner KCS. 2000 Cultural revolution in whale songs. Nature **408**, 537. (10.1038/35046199) 11117730

[18] Nelson DA, Hallberg KI, Soha JA. 2004 Cultural evolution of puget sound white-crowned sparrow song dialects. Ethology **110**, 879-908. (10.1111/j.1439-0310.2004.01025.x)

[19] Fournet MEH, Gabriele CM, Culp DC, Sharpe F, Mellinger DK, Klinck H. 2018 Some things never change: multi-decadal stability in humpback whale calling repertoire on Southeast Alaskan foraging grounds. Sci. Rep. **8**, 13186. (10.1038/s41598-018-31527-x) 30262835 PMC6160409

[20] Rendell L, Whitehead H. 2005 Spatial and temporal variation in sperm whale coda vocalizations: stable usage and local dialects. Anim. Behav. **70**, 191-198. (10.1016/J.ANBEHAV.2005.03.001)

[21] Lachlan RF, Ratmann O, Nowicki S. 2018 Cultural conformity generates extremely stable traditions in bird song. Nat. Commun. **9**, 2417. (10.1038/s41467-018-04728-1) 29925831 PMC6010409

[22] Lachlan RF, Whiten A. 2020 Cultural evolution in non-human animals. In *Oxford bibliographies in evolutionary biology* (ed. D Futuyma). Oxford, UK: Oxford University Press. (10.1093/OBO/9780199941728-0129)

[23] Nakamura M, Uehara S. 2004 Proximate factors of different types of grooming hand-clasp in mahale chimpanzees: implications for chimpanzee social customs. Curr. Anthropol. **45**, 108-114. (10.1086/381007)

[24] van Leeuwen EJC, Cronin KA, Haun DBM, Mundry R, Bodamer MD. 2012 Neighbouring chimpanzee communities show different preferences in social grooming behaviour. Proc. R. Soc. B **279**, 4362-4367. (10.1098/rspb.2012.1543) PMC3479803 22933372

[25] McGrew WC, Marchant LF, Scott SE, Tutin CEG. 2001 Intergroup differences in a social custom of wild chimpanzees: the grooming hand-clasp of the Mahale Mountains. Curr. Anthropol. **42**, 148-153. (10.1086/318441)

[26] Nakamura M, Nishida T. 2013 Ontogeny of a social custom in wild chimpanzees: age changes in grooming hand-clasp at Mahale. Am. J. Primatol. **75**, 186-196. (10.1002/ajp.22098) 23184762

[27] Nishida T. 1988 Development of social grooming between mother and offspring in wild chimpanzees. Folia Primatol. **50**, 109-123. (10.1159/000156335) 3234982

[28] Henrich J, Boyd R. 1998 The evolution of conformist transmission and the emergence of between-group differences. Evol. Hum. Behav. **19**, 215-241. (10.1016/S1090-5138(98)00018-X)

[29] Ron T, McGrew WC. 1988 Ecological assessment for a chimpanzee rehabilitation project in Northern Zambia. Primate Conserv. **9**, 37-41.

[30] Bates D, Mächler M, Bolker B, Walker S. 2015 Fitting linear mixed-effects models using lme4. J. Stat. Softw. **67**, 1-48. (10.18637/jss.v067.i01)

[31] Baayen RH. 2008 Analyzing linguistic data. Cambridge, UK: Cambridge University Press.

[32] Barr DJ, Levy R, Scheepers C, Tily HJ. 2013 Random effects structure for confirmatory hypothesis testing: Keep it maximal. J. Mem. Lang. **68**, 255-278. (10.1016/j.jml.2012.11.001) PMC3881361 24403724

[33] Dobson AJ. 2002 An introduction to generalized linear models. London, UK: Chapman & Hall/CRC.

[34] Lenth R. 2020 Emmeans: estimated marginal means, aka Least-Squares Means. R Package Version 1.4.6.

[35] Nakamura M. 2002 Grooming-hand-clasp in Mahale M Group chimpanzees: implications for culture in social behaviours. In Behavioural diversity in chimpanzees and bonobos (eds C Boesch, G Hohmann, LF Marchant), pp. 71-83. Cambridge, UK: Cambridge University Press.

[36] Wrangham RW et al. 2016 Distribution of a chimpanzee social custom is explained by matrilineal relationship rather than conformity. Curr. Biol. **26**, 3033-3037. (10.1016/j.cub.2016.09.005) 27839974

[37] van Leeuwen EJC. 2021 Data from: Temporal stability of chimpanzee social culture. *Dryad, Dataset*. (10.5061/dryad.6wwpzgmxh) PMC8150033 34034527

[38] van Leeuwen EJC, Mundry R, Cronin KA, Bodamer M, Haun DBM. 2017 Chimpanzee culture extends beyond matrilineal family units. Curr. Biol. **27**, 588-590. (10.1016/j.cub.2017.05.003) 28633025

[39] Whiten A, van Schaik CP. 2007 The evolution of animal ‘cultures’ and social intelligence. Phil. Trans. R. Soc. B **362**, 603-620. (10.1098/rstb.2006.1998) 17255007 PMC2346520

[40] Hoppitt W, Laland KN. 2013 Social learning: an introduction to mechanisms, methods, and models. Oxfordshire, UK: Princeton University Press.

[41] Hoppitt W, Laland KN. 2008 Social processes influencing learning in animals: a review of the evidence. Adv. Study Behav. **38**, 105-165. (10.1016/S0065-3454(08)00003-X)

[42] Galef BG, Laland KN. 2005 Social learning in animals: empirical studies and theoretical models. Bioscience **55**, 489-499. (10.1641/0006-3568(2005)055[0489:SLIAES]2.0.CO;2)

[43] Luncz LV, Boesch C. 2014 Tradition over trend: Neighboring chimpanzee communities maintain differences in cultural behavior despite frequent immigration of adult females. Am. J. Primatol. **76**, 649-657. (10.1002/ajp.22259) 24482055

[44] Boesch C. 2013 Wild cultures: a comparison between chimpanzee and human cultures. Cambridge, UK: Cambridge University Press.

[45] Mercader J, Barton H, Gillespie J, Harris J, Kuhn S, Tyler R, Boesch C. 2007 4,300-Year-old chimpanzee sites and the origins of percussive stone technology. Proc. Natl Acad. Sci. USA **104**, 3043-3048. (10.1073/pnas.0607909104) 17360606 PMC1805589

[46] van Leeuwen EJC, Cronin KA, Haun DBM. 2014 A group-specific arbitrary tradition in chimpanzees (*Pan troglodytes*). Anim. Cogn. **17**, 1421-1425. (10.1007/s10071-014-0766-8) 24916739

[47] Santorelli CJ, Schaffner CM, Campbell CJ, Notman H, Pavelka MS, Weghorst JA, Aureli F. 2011 Traditions in spider monkeys are biased towards the social domain. PLoS ONE **6**, e16863. (10.1371/journal.pone.0016863) 21373196 PMC3044143

[48] Sapolsky RM, Share LJ. 2004 A pacific culture among wild baboons: its emergence and transmission. PLoS Biol. **2**, E106. (10.1371/journal.pbio.0020106) 15094808 PMC387274

[49] van Leeuwen EJC, Cronin KA, Haun DBM. 2018 Population-specific social dynamics in chimpanzees. Proc. Natl Acad. Sci. USA **115**, 11 393-11 400. (10.1073/pnas.1722614115) PMC6233085 30397113

[50] Cantor M, Whitehead H. 2015 How does social behavior differ among sperm whale clans? Mar. Mamm. Sci. **31**, 1275-1290. (10.1111/mms.12218)

[51] Borgeaud C, Sosa S, Bshary R, Sueur C, van de Waal E. 2016 Intergroup variation of social relationships in wild vervet monkeys: a dynamic network approach. Front. Psychol. **7**, 915. (10.3389/fpsyg.2016.00915) 27445890 PMC4914564

[52] Papastamatiou YP, Bodey TW, Caselle JE, Bradley D, Freeman R, Friedlander AM, Jacoby DMP. 2020 Multiyear social stability and social information use in reef sharks with diel fission–fusion dynamics. Proc. R. Soc. B **287**, 20201063. (10.1098/rspb.2020.1063) PMC7575530 32783522

[53] Sapolsky RM. 2006 Social cultures among nonhuman primates. Curr. Anthropol. **47**, 641-656. (10.1086/504162)

[54] Thornton A, Samson J, Clutton-Brock T. 2010 Multi-generational persistence of traditions in neighbouring meerkat groups. Proc. R. Soc. B **277**, 3623-3629. (10.1098/Rspb.2010.0611) PMC2982239 20610431

[55] van Schaik CP, Ancrenaz M, Borgen G, Galdikas B, Knott CD, Singleton I, Suzuki A, Utami SS, Merrill M. 2003 Orangutan cultures and the evolution of material culture. Science **299**, 102-105. (10.1126/Science.1078004) 12511649

[56] van Schaik CP. 2016 The primate origins of human nature. New York, NY: Wiley-Blackwell. (10.1002/9781119118206)

[57] Firth R. 1972 Verbal and bodily rituals of greeting and parting. In The interpretation of ritual (ed. JS La Fontaine), p. 110. London, UK: Routledge.

[58] Duranti A. 1997 Universal and culture-specific properties of greetings. J. Linguist. Anthropol. **7**, 63-97. (10.1525/jlin.1997.7.1.63)

[59] Yerkes RM. 1933 Genetic aspects of grooming, a socially important primate behavior pattern. J. Soc. Psychol. **4**, 3-23. (10.1080/00224545.1933.9921554)

[60] Wrangham RW. 1996 Chimpanzee cultures. Cambridge, MA: Harvard University Press.

[61] Hopper LM, Schapiro SJ, Lambeth SP, Brosnan SF. 2011 Chimpanzees’ socially maintained food preferences indicate both conservatism and conformity. Anim. Behav. **81**, 1195-1202. (10.1016/j.anbehav.2011.03.002) 27011390 PMC4801479

[62] Whiten A, Horner V, de Waal FBM. 2005 Conformity to cultural norms of tool use in chimpanzees. Nature **437**, 737-740. (10.1038/nature04047) 16113685

[63] Vale GL, Davis SJ, van de Waal E, Schapiro SJ, Lambeth SP, Whiten A. 2017 Lack of conformity to new local dietary preferences in migrating captive chimpanzees. Anim. Behav. **124**, 135-144. (10.1016/j.anbehav.2016.12.007) 29200465 PMC5705092

[64] van Leeuwen EJC, Cronin KA, Schütte S, Call J, Haun DBM. 2013 Chimpanzees (*Pan troglodytes*) flexibly adjust their behaviour in order to maximize payoffs, not to conform to majorities. PLoS ONE **8**, e80945. (10.1371/journal.pone.0080945) 24312252 PMC3842352

